# Pediatricians’ Awareness of Pediatric Psoriasis in the Makkah Region, Saudi Arabia

**DOI:** 10.7759/cureus.51985

**Published:** 2024-01-09

**Authors:** Shahad S Alharbi, Amro A Nassif, Rehab B Brnawa, Amjad K Aljuaid, Mai Y Alhajori, Ahmed S Alharbi, Ibrahim Alharbi, Mokhtar Shatla

**Affiliations:** 1 Medicine and Surgery, Umm Al-Qura University, Makkah, SAU; 2 Pediatrics, King Fahad Armed Forces Hospital, Jeddah, SAU; 3 Pediatrics, King Fahad Medical City, Riyadh, SAU; 4 Pediatric Hematology and Oncology, King Fahad Armed Forces Hospital, Jeddah, SAU; 5 Family Medicine, Umm Al-Qura University, Makkah, SAU

**Keywords:** inflammatory diseases, autoimmune diseases, pediatric dermatology, psoriasis diagnosis, psoriasis

## Abstract

Background

Psoriasis is an autoimmune disease characterized by the typical appearance of red, thickened, scaly patches on the skin (plaques). These plaques can vary in size and distribution from person to person. In some individuals, it may affect small areas of skin, while in others, large areas covering their body may be involved.

Patients with psoriasis might be identified by pediatricians before being seen by dermatologists, highlighting the need for pediatricians to be aware and knowledgeable about pediatric psoriasis.

Objective

To evaluate the knowledge and management of pediatric psoriasis among pediatricians and pediatric residents in the Makkah region, Saudi Arabia.

Methods

This was a cross-sectional study using a questionnaire targeted at pediatricians. It inquired about sociodemographic details, knowledge of psoriasis risk factors, diagnosis, management, comorbidity screening, and indications for consulting a dermatologist. The knowledge score cut-off was set at 60%, with scores below this threshold considered indicative of poor knowledge and scores above it reflecting good knowledge.

Results

A total of 139 participants completed the questionnaire; 103 (74.1%) were from Jeddah, 101 (72.7%) were pediatric residents, 118 (84.9%) were general pediatricians, and 18 (15.1%) were in subspecialties. About a third, 49 (35.3%), reported having seen a child with psoriasis. Most participants, 84 (60.4%), had a good knowledge level regarding psoriasis. The vast majority, 119 (85.6%), stated that topical therapies are the first-line treatment; 105 (75.5%) reported being confident in diagnosing psoriasis, and having previously seen a patient with psoriasis was associated with certainty in diagnosis (p-value=0.048); 82 (59%) referred patients to dermatologists. Physician position (p-value=0.049) and being in a general specialty (p-value=0.048) were associated with higher knowledge.

Conclusion

Overall, participants had good knowledge of pediatric psoriasis and its management. However, there is still a need for improvement.

## Introduction

Psoriasis is a chronic inflammatory disease mediated by the immune system, characterized by red, thickened, and scaly patches on the skin (plaques). These plaques can vary in size and distribution from person to person. In some, it may affect small areas of skin, while in others, large areas of their body may be covered. The onset of the disease starts in childhood in up to 35% of adult psoriasis patients [1]. Psoriasis is mainly divided into two age-based groups: pediatric psoriasis, developing before the age of 18 years, and adulthood psoriasis, developing after the age of 18 years [2]. The mean age of onset in pediatric patients is between 8 and 11 years, with an increase in incidence during the second decade of life.

Both men and women can develop psoriasis, although women tend to experience its onset earlier and are more likely to have a positive family history of psoriasis [3]. The age of onset displays a bimodal distribution, peaking 10 years earlier in women and at the ages of 30-39 and 60-69 in men [3]. Psoriasis affects an estimated 60 million people globally, with prevalence rates varying by nation, from 0.05% in Taiwan to 1.88% in Australia [3,4]. Countries with older populations and higher income levels tend to have higher incidences of psoriasis [3]. The general prevalence of psoriasis in Saudi Arabia is around 5.3%. The incidence of childhood psoriasis before the ages of two and ten years is 2% and 10%, respectively [5]. Recently, it has been established that children with psoriasis are at an increased risk of obesity, hyperlipidemia, and metabolic syndrome [6,7].

Pediatric psoriasis is mostly induced by infections and often presents as acute guttate psoriasis. Group A beta-hemolytic streptococcal infection is one risk factor for psoriasis; another is physical and surgical trauma [8]. Additionally, pediatric psoriasis can present as plaque, guttate, erythrodermic, napkin, nail lesions, and arthritis [8]. Autoimmune conditions and allergic skin diseases, such as allergic contact dermatitis, eczema, vitiligo, and alopecia areata, are associated with pediatric psoriasis. However, it may be misdiagnosed as seborrheic dermatitis, neurodermatitis, or balanitis [8]. The clinical presentation of pediatric psoriasis may overlap with other pediatric diseases treated by pediatricians. Therefore, pediatricians may have the opportunity to diagnose children with psoriasis even before they are seen by dermatologists. However, some pediatricians might not be sufficiently knowledgeable or aware to diagnose and manage pediatric psoriasis accurately. This study aimed to evaluate the management of pediatric psoriasis by pediatricians and pediatric residents in Makkah and Jeddah, Saudi Arabia.

## Materials and methods

Study settings and population

This study is a cross-sectional, questionnaire-based study targeting pediatricians, who treat children from birth up to the age of 18 years, working in the Makkah region, including Makkah and Jeddah, Saudi Arabia.

All pediatricians, including residents, specialists, and consultants of all nationalities and both genders, were eligible to participate in the study. Interns, medical students, and paramedics were excluded. Data collection took place from April to October 2021.

Data collection and tool

The questionnaire was designed to be distributed through an online form. The link was sent to participants via email and social media platforms. The form consisted of four major parts: sociodemographic data; knowledge questions about psoriasis risk factors, diagnosis, and management; comorbidity screening of pediatric psoriasis; and indications for dermatology consultation. Data collection took place from April to October 2021. A pilot study involving 139 participants was conducted to ensure the validity of the questionnaire items. This involved modifying the language of items that were poorly understood or difficult to comprehend and then reintroducing them. The process continued until all items were readily understood by the participants.

Ethical considerations

Ethical approval was obtained from the Institutional Review Board of Umm Al-Qura University (approval number: HAPO-02-K012-2021-03-629). Informed consent was acquired before participants started the online form.

Data analysis

The data were collected, sorted, and entered for analysis using IBM SPSS statistical software, version 22. All statistical analyses were performed using two-tailed tests. A p-value of less than 0.05 was considered statistically significant. For knowledge items, each correct answer was scored as one point, and the total summation of the discrete scores from the different items was calculated. A participant with a score less than 60% of the total score was considered to have poor awareness, while those with scores of 60% or more were considered to have good awareness. A descriptive analysis based on frequency and percent distribution was conducted for all variables, including participants’ personal data, job title, specialty, and experience with children who have psoriasis. Additionally, participants’ knowledge and awareness regarding psoriasis, in addition to their certainty of diagnosis and practice, were assessed using frequency tables and graphs. Cross-tabulation was used to assess the distribution of participants’ knowledge levels according to their personal data and practice, in addition to the relationship between their certainty in diagnosing psoriasis and related factors. Relationships were tested using the Pearson chi-square test and the exact probability test for small frequency distributions.

## Results

A total of 139 participants completed the study questionnaire. Of them, 103 (74.1%) were from Jeddah, and 36 (25.9%) were from Makkah. The majority, 101 (72.7%), were residents. As for specialty, 118 (84.9%) had a general specialty, while 21 (15.1%) had subspecialties. The mostA total of 49 (35.3%) participants reported having seen a child with psoriasis. See the results detailed in Table 1.

**Table 1:** Personal data of study participants in Saudi Arabia. N: Number of the participants; (%): Proportion of the participants; NICU: Neonatal Intensive Care Unit.

Variable	Groups	N	%
Region	Jeddah	103	74.1%
	Makkah	36	25.9%
Position	Consultant	16	11.5%
	Resident R1	43	30.9%
	Resident R2	25	18.0%
	Resident R3	10	7.2%
	Resident R4	23	16.5%
	Specialist	22	15.8%
Specialty	General	118	84.9%
	Subspecialties	21	15.1%
	General Pediatrics	55	88.8%
	NICU	2	3.2%
	Allergy immunology	1	1.6%
	Endocrinology	1	1.6%
	ER	1	1.6%
	Infectious diseases	1	1.6%
	Metabolic diseases	1	1.6%
Have you ever seen a child with psoriasis?	Yes	49	35.3%
	No	90	64.7%

The participants were given eight knowledge statements about psoriasis and asked to indicate whether they were true or false. In all the statements, 'true' was indicated by more than half of the participants. When asked about the first-line treatment of psoriasis, the most commonly reported response was topical therapies, indicated by 119 (85.6%) participants. The results of the knowledge statements are shown in Table 2.

**Table 2:** Knowledge regarding psoriasis among study participants in Saudi Arabia. True, N(%): Frequency and proportion of the participants answering true. False, N(%): Frequency and proportion of the participants answering false.

Knowledge items	True, N (%)	False, N (%)
Psoriasis can begin at any age, including infancy	120 (86.3%)	19 (13.7%)
Up to 90% of patients have a family history of psoriasis	87 (62.6%)	52 (37.4%)
Guttate psoriasis is seen more commonly in children and young adults	85 (61.2%)	54 (38.8%)
Chronic plaque psoriasis is the most common presentation	82 (59%)	57 (41%)
Psoriasis is described as pink to red papules and plaques with thick white scales	126 (90.6%)	13 (9.4%)
The distribution of lesions involves the scalp and the extensor aspect of the elbows and knees	127 (91.4%)	12 (8.6%)
A skin injury and scratching can trigger psoriasis	96 (69.1%)	43 (30.9%)
Sudden appearance of generalized numerous monomorphic psoriasis papules is often triggered by streptococcal infection	97 (69.8%)	42 (30.2%)

**Figure 1:**
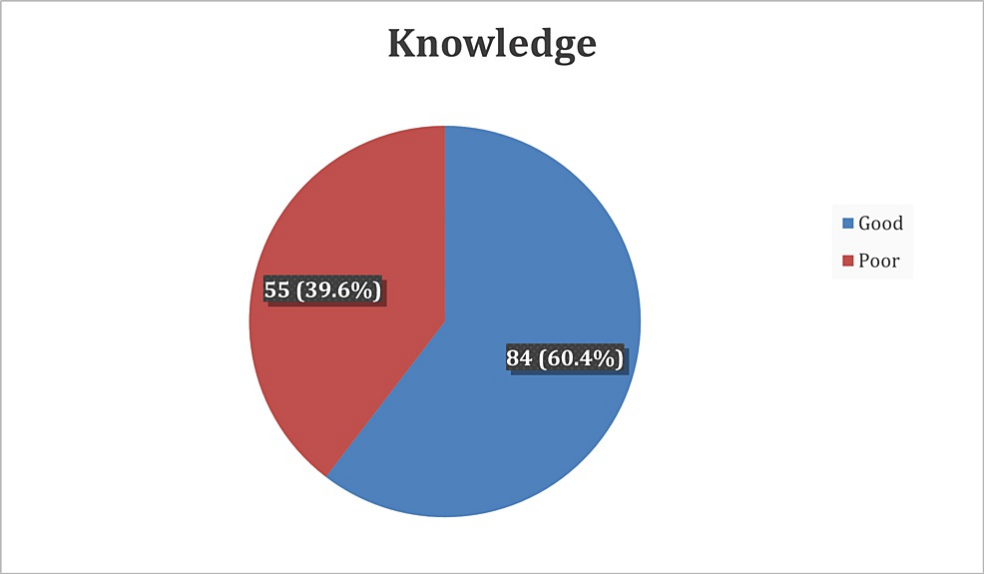
Overall knowledge level regarding psoriasis among study participants in Saudi Arabia.

The knowledge items were computed, with 84 (60.4%) of the correctly answered items considered indicative of a good level of knowledge (Figure 1). 

The majority of participants, 105 of 139 (75.5%), reported certainty to a very high degree regarding their ability to diagnose psoriasis. The most-seen comorbidities with childhood psoriasis were identified as psoriatic arthritis, 83 (59.7%). A total of 82 (59%) participants reported referring all cases of psoriasis to dermatologists. The practice items and referral reasons from the survey are shown in Table 3.

**Table 3:** Participants’ practice regarding psoriasis in Saudi Arabia. N: Number of participants; (%): Proportion of the participants.

Practice items		N	%
Degree of certainty of the clinical diagnosis of psoriasis	Very certain	12	8.6%
	Certain	93	66.9%
	Uncertain	30	21.6%
	Very uncertain	4	2.9%
Do you assess the severity of psoriasis cases?	Yes	42	30.2%
	No	33	23.7%
	Don’t know how to assess	64	46.0%
Do you screen for co-morbidities?	Yes	66	47.5%
	No	73	52.5%
Co-morbidities seen with childhood psoriasis?	Psoriatic arthritis	83	59.7%
	Diabetes mellitus	33	23.7%
	Hyperlipidemia	29	20.9%
	Others	26	18.7%
	Hypertension	24	17.3%
	Obesity	21	15.1%
	None	36	25.9%
Do you refer all cases of psoriasis to dermatologists?	Yes	82	59.0%
	No	34	24.5%
	Only severe cases	23	16.5%
Reason for referring patients to dermatologists?	Initiate therapy in line with guidelines	56	54.9%
	To confirm the diagnosis	35	34.3%
	Screen for co-morbidities	7	6.9%
	Others	4	3.9%

The level of knowledge about psoriasis was statistically tested to find significant associations with position, specialty, and other variables. The position of the physician was significantly associated with the level of knowledge regarding psoriasis (p-value = 0.049). Physicians categorized under the specialty of 'general practice or having a subspecialty' also showed a statistically significant association with the level of knowledge (p-value = 0.048). The results of the significance of other variables are shown in Table 4.

**Table 4:** Distribution of participants’ knowledge regarding psoriasis by their personal data and practice. N (%): Frequency and Proportion of the Participants P: Pearson's chi-squared test; $: Exact probability test; * p-value < 0.05 (significant) A p-value is considered significant if it is less than 0.05.

	Knowledge		
	Good N (%)	Poor N (%)	P-value
Position			
Consultant	9 (56.3%)	7 (43.8%)	0.049*
Specialist	17 (77.3%)	5 (22.7%)	
Resident	58 (57.4%)	43 (42.6%)	
Specialty			
General	74 (62.7%)	44 (37.3%)	0.048*
Subspecialties	10 (47.6%)	11 (52.4%)	
Have you ever seen a child with psoriasis?			
Yes	29 (59.2%)	20 (40.8%)	0.824
No	55 (61.1%)	35 (38.9%)	
Degree of certainty of the clinical diagnosis of psoriasis			
Very certain	5 (41.7%)	7 (58.3%)	0.169^$^
Certain	57 (61.3%)	36 (38.7%)	
Uncertain	21 (70%)	9 (30%)	
Very uncertain	1 (25%)	3 (75%)	
Do you assess the severity of psoriasis cases?			
Yes	28 (66.7%)	14 (33.3%)	0.426
No	21 (63.6%)	12 (36.4%)	
Don’t know how to assess	35 (54.7%)	29 (45.3%)	
Do you screen for co-morbidities?			
Yes	36 (54.5%)	30 (45.5%)	0.152
No	48 (65.8%)	25 (34.2%)	
Do you refer all cases of psoriasis to dermatologists?			
Yes	51 (62.2%)	31 (37.8%)	0.865
No	20 (58.8%)	14 (41.2%)	
Only severe cases	13 (56.5%)	10 (43.5%)	

The relationship between participants' certainty in diagnosing psoriasis and their personal data and practice was tested to determine statistical association. While 41 (83.7%) participants who had seen a child with psoriasis showed certainty in diagnosing psoriasis, 64 (71.1%) of those who had never seen a case expressed similar certainty (p-value = 0.048). While other factors did not show statistical significance, the responses varied across the different groups. See the results in Table 5.

**Table 5:** Distribution of participants’ certainty regarding psoriasis diagnosis by their personal data and practice. N (%): Frequency and Proportion of the Participants P: Pearson's chi-squared test; $: Exact probability test; * p-value < 0.05 (significant) A p-value is considered significant if it is less than 0.05.

	Degree of certainty of the clinical diagnosis of psoriasis				
	Very certain N (%)	Certain N (%)	Uncertain N (%)	Very uncertain N (%)	P-value
Position					
Consultant	0 (0%)	12 (75%)	4 (25%)	0 (0%)	0.459^$^
Specialist	0 (0%)	16 (72.7%)	5 (22.7%)	1 (4.5%)	
Resident	12 (11.9%)	65 (64.4%)	21 (20.8%)	3 (3%)	
Specialty					
General	12 (10.2%)	77 (65.3%)	26 (22%)	3 (2.5%)	0.418^$^
Subspecialties	0 (0%)	16 (76.2%)	4 (19%)	1 (4.8%)	
Have you ever seen a child with psoriasis?					
Yes	3 (6.1%)	38 (77.6%)	8 (16.3%)	0 (0%)	0.048*^$^
No	9 (10%)	55 (61.1%)	22 (24.4%)	4 (4.4%)	
Do you screen for co-morbidities?					
Yes	7 (10.6%)	45 (68.2%)	13 (19.7%)	1 (1.5%)	0.656
No	5 (6.8%)	48 (65.8%)	17 (23.3%)	3 (4.1%)	
Do you refer all cases of psoriasis to dermatologists?					
Yes	5 (6.1%)	55 (67.1%)	20 (24.4%)	2 (2.4%)	0.284^$^
No	6 (17.6%)	21 (61.8%)	5 (14.7%)	2 (5.9%)	
Only severe cases	1 (4.3%)	17 (73.9%)	5 (21.7%)	0 (0%)	

## Discussion

This study evaluates the management of pediatric psoriasis by pediatricians and pediatric residents in Makkah and Jeddah, Saudi Arabia. About three-quarters of participants reported being confident in their ability to diagnose psoriasis, and those who had previously seen a child with psoriasis were more confident than those who had never seen a child with psoriasis (p=0.048), highlighting the importance of exposure and experience in making the correct diagnosis and better managing the disease. Exposure leads to an accurate understanding of the patient's health problem, enhancing accurate clinical decision-making and successful patient outcomes [9]. Psoriasis is mainly clinically diagnosed; therefore, a full-body inspection is required to diagnose and correctly determine the disease's severity [1]. A study conducted in Germany showed that pediatricians who felt certain about their diagnosis were more likely to perform a whole-body examination [1]. Nevertheless, only a third of our participants reported having assessed the severity of psoriasis cases. This might be attributed to the low percentage of pediatricians who saw children with psoriasis since most (64.7%) participants had never seen a child with psoriasis. However, another study showed that less than 5% of pediatricians declared they are assessing severity [10], compared to 30.2% in our study. This might be explained by our participants having a good knowledge of using the different scores for evaluating the severity of pediatric psoriasis. While the Psoriasis Area Severity Index (PASI) is commonly used to assess psoriasis severity, it is complex and time-consuming, affecting its use in clinical practice [11]. Therefore, a simple alternative that is more sensitive to the dynamic changes of psoriasis, the Optimal Psoriasis Assessment Tool (OPAT), was introduced. It was found that while PASI requires 16 measurements and 13 calculations, OPAT requires only body surface area and one patient-reported outcome [11], making it a convenient but still effective tool.

Screening for comorbidities is vital in psoriasis management since children with psoriasis have a two-fold more significant risk of obesity, type II diabetes, hypertension, and non-alcoholic fatty liver disease and a significantly increased risk of hyperlipidemia [7,12]. Despite that, more than half of our participants did not screen for comorbidities. A previous study showed that pediatricians have a low screening rate for comorbidity and dermatology referrals [10]. This study showed that pediatricians consider psoriatic arthritis to be the most common comorbidity, which is consistent with another study showing that pediatricians are more likely to screen for psoriatic arthritis than other comorbidities [1]. Perhaps this focus is due to a higher knowledge of psoriatic arthritis than metabolic comorbidities. Furthermore, metabolic syndrome is considered more often in adults than children. An expert panel on psoriasis comprised of multidisciplinary professionals established recommendations for screening comorbidities in children with psoriasis [13]. This panel recommended that clinicians screen annually for obesity and overweight, starting at the age of two, and every three years for diabetes, starting at the age of 10, using fasting glucose measurements. Clinicians should screen patients between the ages of 9 and 11 and 17 and 21, as well as patients at risk of cardiovascular diseases, for dyslipidemia. Biomarkers have the potential to improve the evaluation and management of psoriatic disease. There is still limited data available to validate candidate biomarkers for the different clinical forms of the disease. It could be used in clinical practice to assess disease severity or as endpoints in studies of therapeutic interventions, and it could also use genetic variants as predictive risk factors for the development of psoriatic disease. Moreover, detecting changes in gene expression that correlate with responses to biological therapies is a promising field for developing new biomarkers that will help select the most effective therapy for individual patients. Clinicians should conduct yearly hypertension screenings for patients starting at age three, using age, sex, and height reference charts [13,14]. For obese or overweight children aged 9 to 11 who have additional risk factors, Alanine aminotransferase tests are recommended to screen for fatty liver disease [15,16]. Pediatric psoriasis patients should undergo a physical exam and a targeted systemic evaluation to check for arthritis [14]. Finally, the panel recommended annual check-ups for depression and anxiety and yearly substance abuse screening from the age of 11 [13]. The screenings are designed to guide the proper holistic management of patients with a multidisciplinary approach. The American Academy of Dermatology recommends topical corticosteroids as monotherapy for the short-term treatment of localized psoriasis in pediatric patients [17]. A majority of the participants (85.6%) considered topical corticosteroids the first line of treatment for mild psoriasis. Referring patients to a dermatologist is beneficial for diagnosis and management. However, 34.4% of our study participants stated that their reason for referring was to confirm the diagnosis. This is in contrast to another study, which showed that more than 80% of participants referred their patients for that reason [1]. This difference might indicate a higher confidence in diagnosing psoriasis among our study participants.

Our study findings underline that targeted training sessions for pediatricians on the use of systemic therapies, severity assessment, comorbidities screening guidelines in pediatric populations with psoriasis, and intensified interdisciplinary cooperation with dermatologists are needed [1,18]. These could improve the certainty of the diagnosis, identification of risk factors and associated comorbidities, and quality of care [18]. Some of our study limitations include the small sample, which might limit the generalization of results. There was unequal distribution regarding the regions represented. Most participants were from Jeddah, with a percentage of 86.3%, compared to the percentage from Makkah of 25.9%. In addition, most of the participants in this study were juniors, with a percentile of 30.9% for R1 residents and 18% for R2 residents. Inequality of levels may impact reflection on the results.

## Conclusions

This study showed that participants had a good level of knowledge and confidence in diagnosing psoriasis overall. However, their screening habits for associated comorbidities were poor, albeit better than those reported in similar studies. Pediatricians should be aware of pediatric dermatology diseases, such as pediatric psoriasis and its associated comorbidities. These conditions can significantly affect a child's quality of life. Pediatricians are often the first to examine children with these diseases and play a crucial role in guiding the next phases of care. In such situations, a dermatological consultation should be considered. Early detection and intervention of psoriasis in children significantly prevent serious complications and improve their quality of life. We found that general pediatricians and pediatric residents possess good knowledge about pediatric psoriasis, its complications, and basic management. Therefore, to further improve and update information regarding psoriasis in pediatric care, we suggest initiatives such as increasing educational activities, promoting dermatology clinic attendance, and encouraging regular reading and research among our physicians.
